# Population trends related to injury from explosive munitions in Lao PDR (1964–2008): a retrospective analysis

**DOI:** 10.1186/s13031-018-0171-z

**Published:** 2018-08-22

**Authors:** Stacey E. Pizzino, Samuel Hundessa, Vinu Verghis, Mark Griffin, Jo Durham

**Affiliations:** School of Public Health, Faculty of Medicine, The University of Queensland, Brisbane, Australia

**Keywords:** Landmines, Explosive remnants of war, Unexploded ordnance, Conflict, War, Lao PDR

## Abstract

**Background:**

The presence of landmines and explosive remnants of war (ERW) including unexploded ordnance (UXO) poses a serious public health risk for populations living in conflict-affected and contaminated areas. Current analysis, however, provides only a partial view of the burden. In this study, we examined the multivariable relationship between year of injury, activity at the time of the incident, case fatalities and casualty rates in order to provide decision-makers with a more fine-grained understanding of landmines and ERW injuries in the Lao PDR.

**Methods:**

Using data from a retrospective, national household survey, frequency tables, logistic and Poisson regressions were performed using STATA 13 to predict the case fatality and population-standardized incidence rates for ERW casualties.

**Results:**

The findings indicated that most casualties were male (86.75%), with the majority of incidents (74.7%) occurring during the conflict period (1964–1979). The odds of death for the conflict period was 1.5 times that of the post-conflict period (1980–2008). The highest odds of death during the conflict period was associated with big bombs (OR = 1.38, 95% CI: 1.243–1.522, *p* < 0.01), and landmine injuries were more common during conflict compared to the post-conflict period (IRR = 1.42, 95% CI: 1.368–1.477, p < 0.01). Post conflict, cluster munitions had the highest incidence rate for death or injury (IRR = 1.07, 95%CI: 1.006–1.143, *p* = 0.03). Scrap collection which is often the target of mine risk education and thought to be one of the main activities at time of injury had the second lowest incidence rate of the activities related to incident during post-conflict period.

**Conclusions:**

As the first study of this nature in Lao PDR, this research provides information essential for planning services and prevention. This study suggests more effort needs to be directed towards addressing the geographical regions and population subgroups experiencing increased casualty numbers and odds of death. Further research is required to improve the documentation and understanding of the health and socio-economic consequences of landmine and ERW injuries.

## Background

Following violent conflict, the continued presence of landmines and explosive remnants of war (ERW)^1^ including unexploded ordnance (UXO) poses a serious public health risk for populations living in conflict-affected and contaminated areas [1–3]. Currently, more than 65 countries are thought to be contaminated, including Lao People’s Democratic Republic (PDR), Afghanistan, Lebanon, and Sudan [4]. While the number of reported deaths in post-conflict contexts has been steadily declining, the number of survivors has increased with an estimated 226,000–358,000 survivors in countries with significant ERW contamination, many of which are civilians [5]. However, these figures are likely to be underestimated as most injuries occur in low/lower-middle-income countries where injury surveillance systems are often inadequate and incomplete [6, 7].

Landmines, an explosive weapon designed to incapacitate or kill victims via pressure activation, trip wires or remote detonation, can be laid by hand, artillery or via aircraft, and typically result in catastrophic lower limb injuries [8]. Whilst landmines in South East Asia have long been associated with Cambodia, Lao PDR also has a significant history of landmine contamination with landmine-related casualties peaking during the Second Indo-China War (1964–1973) [9]. Today UXO, particularly cluster munition remnants, are the main cause of injury within the region, with Lao PDR thought to have one of the highest numbers of cluster munition casualties globally [10]. While cluster munitions have been used in armed conflict since 1943, and in more than 35 countries, including in Syria, Yemen, South Sudan and Ukraine [11], the most extensive deployment of cluster munitions occurred during the second Indo-China conflict (1964–1973). During this period the United States of America dropped at least 1.5 million tonnes of ordnance over Lao PDR [9]. This ordnance consisted of primarily cluster munitions, an aerial bomb containing numerous explosive sub-munitions of which an estimated 80 million failed to detonate, resulting in approximately 8,470km^2^ of ERW-contaminated land across Lao PDR [12, 13]. Most of the contamination within Lao PDR is in rural areas where 61% of the Lao population reside, reliant on subsistence agricultural practices [14].

Whilst injuries as a result of landmines have been highlighted within international literature, much less has been written about cluster munitions. Cluster munitions are either spherical or cylindrical shaped allowing them to release their explosive energy, and the subsequent particle wave, in a radiating manner [15]. This makes their mechanism of injury twofold: first, there is a blast and temperature wave, followed by a particle wave consisting of preformed shrapnel and the debris of the proximate environment [15]. Their design means they have no defusing mechanism, thus if they fail to detonate on impact they remain fused until physically triggered (i.e., by being hit or moved) [16]. When triggered, injuries can include traumatic amputation, traumatic brain injuries and multiple fragment wounds to the torso, hands and face [16–20]. Secondary effects can include infections and auditory impairments such as tinnitus and deafness [19].

Landmines and ERW lie in close proximity to the surface, and when hit or moved in the course of routine agricultural or domestic activities may explode causing severe injury [21, 22]. Beyond individual casualty, landmines and ERW cause long-term socioeconomic effects. Disabilities arising from severe non-fatal injuries exacerbates poverty in an already vulnerable population, while contamination can act as a barrier to opening new land for agriculture and can prevent the development of basic infrastructure, including water, sanitation and essential services such as hospitals, health centres and schools [23].

Most reports on landmine and ERW injury use frequency measures. While these can provide valuable information, particularly in terms of detecting trends, on their own they provide policy-makers with a partial view of the problem. They do not, for example, give a picture of the proportion of people affected in a certain area, nor do they help to identify risk-factors. In this study, we examined the multivariable relationship between year of injury, activity at the time of the incident, case fatalities and casualty rates using data from a national, retrospective survey. This is significant, as the first study of this kind in Lao PDR it provides information for planning services and prevention.

## Methods

### Study area

This study was conducted in Lao PDR which is located in South East Asia, bordered by Cambodia, Thailand, Myanmar, Vietnam and China. Lao PDR encompasses 18 provinces and 141 districts. According to the national census in 2015, Lao PDR has a population of 6,492,228 [24].

### Data material

The present study used data collected as part of the first *National UXO Victim and Accident Survey* [9]. This retrospective, household survey was undertaken by the National Regulatory Authority (NRA) in Lao PDR and collected data on ERW injuries from the beginning of 1964 [9]. In total, the NRA survey covered 139 of the 141 Districts, and 17 of the 18 Provinces [9]. Trained enumerators visited 9583 different villages collecting data from village leaders and the available historical records in each village, and where possible, interviewed survivors and/or the families of the UXO casualties or an eyewitness [9]. Information collected included the date and location of the incident, victim demographics, circumstances of the incident (e.g. casualty’s activity at the time of incident, type of explosive device that caused the incident) and nature of the injury. For the purpose of the survey, a casualty was defined as someone, alive or dead, who had suffered an ERW accident.

To adjust for the heterogenous population densities among different provinces/district, we used the district-level population data of the 1995 census available from the National Statistical Bureau of Lao PDR, and 1985 population data accessed from a previous publication [25]. The 1995 census data were used for this research, rather than the more recent 2005 or 2015 censuses, because these were the closest to the time of most incidents.

Prior to starting the statistical analysis, the UXO data were cleaned and recoded to avoid any redundancy among the UXO types and cause of incident classifications. The UXO data were matched to the population dataset in R Software version 3.2.2. After checking for missing values for each variable, observations with missing values were allowed to be removed from further analysis as their proportions per year intervals were negligible (i.e., less than 4%).

### Analysis

The statistical analyses were performed after stratifying the data by time interval of incident as during conflict (1964–1979) and post-conflict (1980–2008). For each of the strata, logistic regression models were developed to estimate the case fatality (the number of deaths compared to the number of injuries) using the casualties’ gender, activity at time of incident, UXO type, year of incident, and location of the incident as predictors. For conducting the Poisson regression analysis, the incidents (death/injury) were aggregated to provincial or district-level and used in the Poisson regression model with the above-mentioned predictors to estimate the incidence rate of the casualties. All the statistical analyses were performed using STATA version 13.1. The appropriateness of these models was confirmed by diagnostic checking that considered collinearity (through determining the VIF of an equivalent linear regression model), the absence of specification errors (using a link test), linearity (using a Box-Tidwell model), and the absence of outliers (through a residual plot).

### Ethics

The initial survey was approved by the NRA and collected in face-to-face interviews with informed consent. The data were collected for public use and made available to the research team for the purpose of this study with the approval of the NRA. All data used by the study team were de-identified. For these reasons, additional ethical approval was not sought.

## Results

### Demographics

**Fig. 1:**
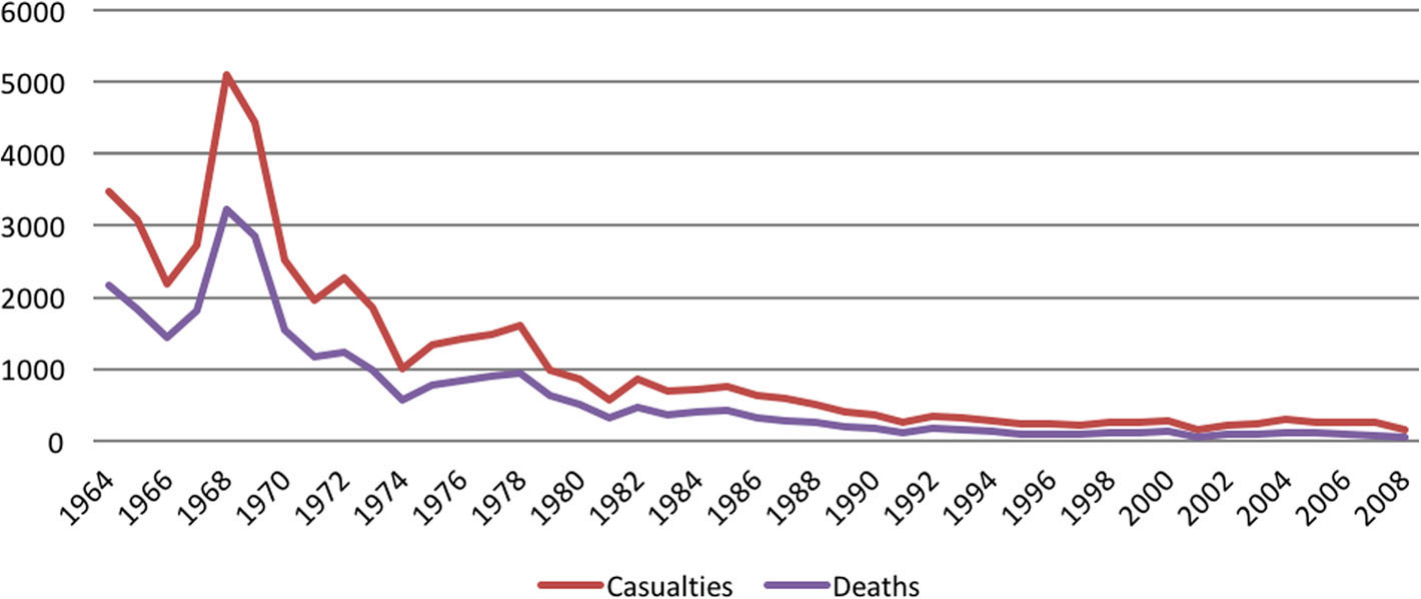
The number of casualties and deaths as a function of time

The survey identified 44,874 people who were injured or killed by ERW. Most casualties were male (86.75%), with the majority of incidents occurring between 1964 and 1979 (74.47%). Soldiers (45.03%), and farmers (34.53%) represented the most common occupations for casualties, with students (6.59%) and children (6.35%) also experiencing higher casualty rates. Most casualties died as a result of the ERW incident (58.34%). Figure 1 shows the number of casualties and deaths as a function of time.

**Fig. 2:**
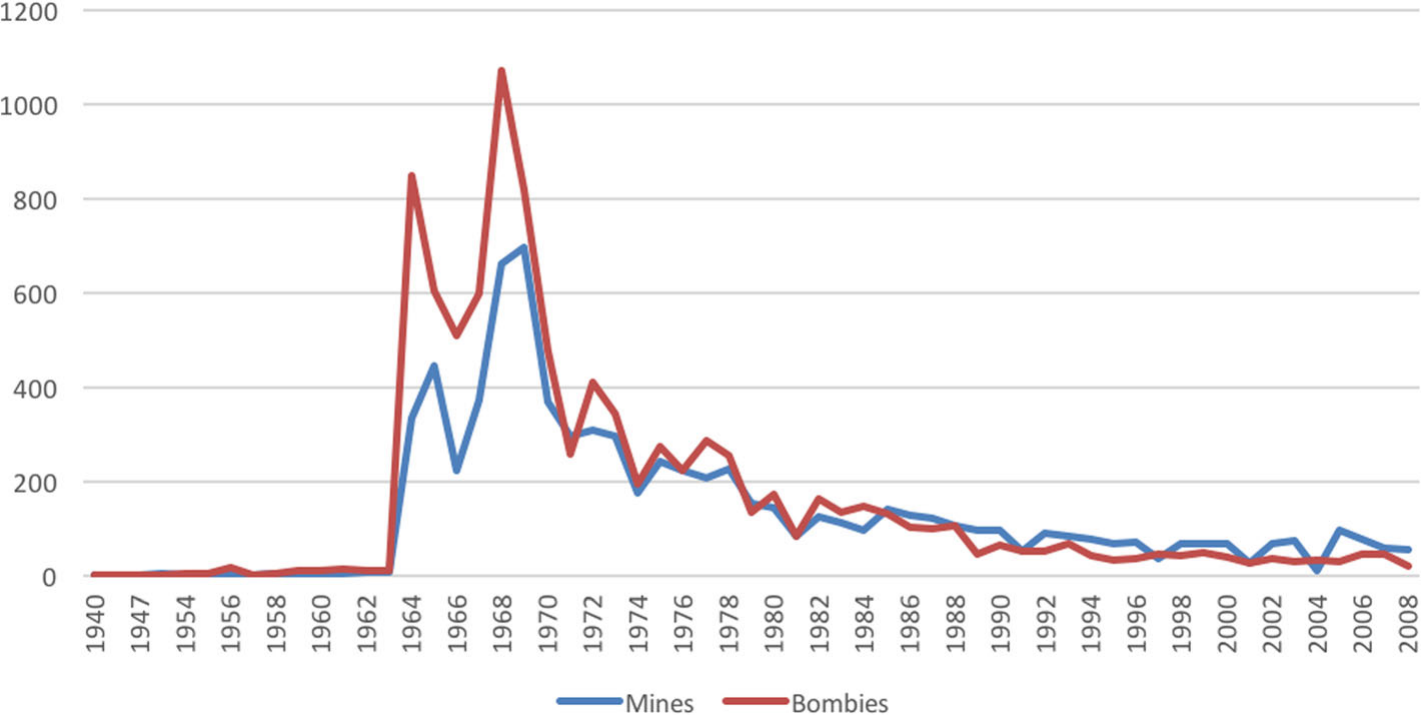
Number of Mine and Bombie Incidents Across Time

The most common activities at the time of the incident were: travelling (17.04%); warfare (16.1%); and agricultural practices (i.e., collecting food, water or wood, farming/gardening, and digging) (15.85%). The most common explosive causative agents identified were landmines (18.73%) and cluster munitions (bombies) (15.96%). Figure 2 shows the number of incidents involving landmines and bombies as a function of time.

The provinces with the largest number of incidents were Savannakhet (24.95%), Xieng Khouang (14.23%) and Huaphan (7.81%). Summary statistics of this dataset can be found in Table 1.

**Table 1:** Summary statistics of the UXO dataset (including the absolute number and proportion of victims in each response category, and the levels of missing data)

Variable	Response Category	Absolute Number	Proportion
Gender	Female	5939	13.23
	Male	38,928	86.75
	Missing	7	0.02
Year	1964–1979	33.416	74.47
	1980–2008	11,453	25.53
Activity	Bombed by plan	4889	10.89
	Bystander, doing nothing	4740	10.56
	Collecting food, water or wood	2696	6.01
	Disturbed by animals	1614	3.60
	Gun violence	4358	9.71
	Making a fire	2308	5.14
	Playing (children)	1103	2.46
	Scrap Collection	170	0.38
	Tampering	1806	4.02
	Travelling	7648	17.04
	Warfare	7344	16.37
	Farming or Gardening/Digging	4416	9.84
	Suicide	15	0.03
	Missing	1767	3.94
Device type	Big bomb	4266	9.51
	Small bomb	1158	2.58
	Bombie	7163	15.96
	Mine	8405	18.73
	Mortar	2431	5.42
	Rocket	1936	4.31
	Fuse	233	0.52
	Artillery shell	4965	11.06
	White phosphor bomb	172	0.38
	Grenade	3674	8.19
	Other	8183	18.0
	Unknown	2084	4.64
	Missing	234	0.52
Province	Vientiane Capital	967	2.15
	Phongsaly	437	0.97
	Luang Namtha	1014	2.26
	Oudomxay	2590	5.77
	Bokeo	805	1.8
	Luang Prabang	3235	7.1
	Huaphan	3504	7.81
	Xayabouly	1167	2.6
	Xiengkhouang	6552	14.4
	Vientiane province	1411	3.14
	Bolikhamxay	1197	2.67
	Khammuane	2773	6.1
	Savannakhet	11,197	24.95
	Saravan	3523	7.85
	Sekong	1161	2.59
	Champasack	1563	3.48
	Attapeu	2080	4.64
	Missing	33	0.1
Outcome	Death	26,178	58.34
	Injured	18,683	41.63
	Missing	13	0.03
Total		44,874	100.0

### Overall (1964–2008)

Table 2 shows the results of the multivariate logistic regression for the association between case fatality and activity at time of incident, gender of casualty, UXO type and the provinces across time. The results indicate that odds of death differ by activities at the time of incident, UXO type, the provinces and year category but not by gender. Gun wounds (OR = 1.18, 95% CI: 1.068–1.294, *p* < 0.01) and suicide (OR = 5.91, 95% CI: 1.326–26.339, *p* = 0.02) were associated with higher odds of death while collecting food, water or wood, disturbance by animals, making fire, playing, tampering, travelling, warfare, farming and digging were associated with lower odds of death (refer Table 2).

**Table 2:** Logistic regression analysis of case fatality across all time periods

		OR	*p*-value	95% CI	
Year	1965–1979	1.55	0.00	1.479	1.630
	^a^1980-2008	1.00			
Gender	Male	1.01	0.82	0.946	1.072
	^a^Female	1.00			
Cause of Accident	Bombed by plane	0.99	0.90	0.900	1.097
	Collecting Food Water or Wood	0.77	0.00	0.692	0.847
	Disturbed by animals	0.66	0.00	0.582	0.738
	Gun Violence	1.18	0.00	1.068	1.294
	Making Fire	0.39	0.00	0.348	0.430
	Playing	0.74	0.00	0.646	0.851
	Scrap Collection	1.31	0.11	0.937	1.845
	Tampering	0.57	0.00	0.506	0.648
	Travelling	0.83	0.00	0.768	0.902
	Warfare	0.69	0.00	0.635	0.749
	Farming or Gardening	0.80	0.00	0.732	0.876
	Suicide	5.91	0.02	1.326	26.339
	^a^Doing nothing/ Bystander	1.00			
UXO Type	Bombie	0.87	0.00	0.804	0.931
	Artillery shell	0.84	0.00	0.778	0.910
	Big Bomb	1.34	0.00	1.215	1.468
	Small Bomb	1.04	0.57	0.905	1.200
	Grenade	0.72	0.00	0.662	0.784
	Mortar	0.72	0.00	0.650	0.791
	Rocket	0.87	0.01	0.781	0.971
	Fuse	0.32	0.00	0.236	0.425
	White Phosphor Bomb	0.58	0.00	0.423	0.794
	Other	0.84	0.00	0.785	0.906
	Unknown	1.42	0.00	1.268	1.580
	^a^Landmine	1.00			
Provinces	Attapeu	2.20	0.00	1.761	2.736
	Bokeo	1.24	0.08	0.971	1.592
	Bolikhamxay	1.03	0.81	0.817	1.295
	Champasack	1.30	0.02	1.042	1.630
	Huaphan	1.31	0.01	1.066	1.620
	Khammuane	1.44	0.00	1.161	1.784
	Luang Namtha	1.28	0.05	1.006	1.617
	Luang Prabang	1.00	1.00	0.810	1.235
	Oudomxay	1.22	0.07	0.983	1.517
	Saravan	1.70	0.00	1.380	2.103
	Savannakhet	2.03	0.00	1.654	2.484
	Sekong	1.76	0.00	1.391	2.223
	Vientiane Capital	0.35	0.00	0.273	0.448
	Vientiane province	1.17	0.18	0.931	1.469
	Xayabouly	0.78	0.04	0.620	0.989
	Xiengkhouang	1.10	0.36	0.896	1.352
	^a^Phongsaly	1.00			

^a^Reference group

A statistically significant difference in odds of death was observed between provinces. The odds of death were higher in Attapeu (OR = 2.20, 95% CI: 1.761–2.736, *p* < 0.01), Khammuane (OR = 1.44, 95% CI: 1.161–1.784, *p* < 0.01), Savannakhet (OR = 2.03, 95% CI: 1.654–2.484, *p* < 0.01), Sekong (OR = 1.76, 95% CI: 1.391–2.223, *p* < 0.01) and Saravan (OR = 1.70, 95% CI: 1.380–2.103, *p* < 0.01), while Vientiane Capital had lower odds of death (OR = 0.35, 95% CI: 0.273–0.448, *p* < 0.01).

This study also showed a statistically significant difference in odds of death during and after the war. The odds of death during the war (1964–1979) was 1.5 times higher than the odds of death post-conflict (1980–2009).

### Strata 1: During conflict (1964–1979)

The findings indicate that males are expected to have an incidence rate of death/injury (the outcome) 4.34 times greater than females (IRR = 4.34, 95% CI: 4.203–4.479, *p* < 0.01). The significant causes of incident during 1965–1979 include warfare (IRR = 3.35, 95% CI: 3.209–3.497, *p* < 0.01), bombed by plane (IRR = 3.28, 95% CI: 3.134–3.430, *p* < 0.01), travelling (IRR = 3.10 95% CI: 2.974–3.240, *p* < 0.01), and gun violence (IRR = 2.0, 95% CI: 1.908–2.106, *p* < 0.01). While animal disturbance, making fire, tampering, farming, gardening and digging, scrap metal collection, and playing were associated with lower incidence rate, there was no significant difference in the incidence rate between males and females. Among the recorded explosive device types, the analysis shows that landmines had an incidence rate 1.42 times higher for the death/injury compared to the other explosive device types (Table 3).

**Table 3:** Poisson regression analysis of case fatality during the conflict period (1965–1979)

		IRR	Std. Err.	*p*-value	95% CI	
Gender	Male	4.34	0.07	0.00	4.203	4.479
	^a^Female	1.00				
Cause of Accident	Bombed by plane	3.28	0.08	0.00	3.134	3.430
	Collecting Food Water or Wood	0.99	0.03	0.69	0.932	1.048
	Disturbed by animals	0.75	0.03	0.00	0.704	0.808
	Gun Violence	2.00	0.05	0.00	1.908	2.106
	Making Fire	0.83	0.03	0.00	0.774	0.883
	Playing	0.36	0.02	0.00	0.324	0.395
	Scrap Collection	0.11	0.01	0.00	0.081	0.138
	Tampering	1.02	0.03	0.53	0.957	1.090
	Travelling	3.10	0.07	0.00	2.974	3.240
	Warfare	3.35	0.07	0.00	3.209	3.497
	Farming or Gardening/digging	0.84	0.03	0.00	0.791	0.895
	Suicide	0.86	0.03	0.00	0.804	0.916
	^a^Doing nothing /Bystander	1.00				
UXO type	Landmine	1.42	0.03	0.00	1.368	1.477
	Artillery shell	0.83	0.02	0.00	0.790	0.862
	Big Bomb	0.94	0.02	0.01	0.902	0.982
	Small Bomb	0.27	0.01	0.00	0.256	0.295
	Grenade	0.57	0.01	0.00	0.542	0.598
	Mortar	0.42	0.01	0.00	0.394	0.441
	Rocket	0.37	0.01	0.00	0.347	0.390
	Fuse	0.11	0.01	0.00	0.091	0.130
	White Phosphor Bomb	0.08	0.01	0.00	0.070	0.099
	Other	1.21	0.02	0.00	1.163	1.259
	Unknown	0.41	0.01	0.00	0.387	0.435
	^a^Bombie					
Provinces	Attapeu	5.94	0.37	0.00	5.251	6.709
	Bokeo	3.30	0.23	0.00	2.873	3.796
	Bolikhamxay	1.46	0.10	0.00	1.278	1.668
	Champasack	0.63	0.04	0.00	0.556	0.716
	Huaphan	2.52	0.15	0.00	2.237	2.835
	Khammuane	1.94	0.12	0.00	1.718	2.187
	Luang Namtha	2.27	0.15	0.00	1.987	2.594
	Luang Prabang	1.69	0.10	0.00	1.504	1.908
	Oudomxay	3.27	0.20	0.00	2.898	3.681
	Saravan	3.26	0.20	0.00	2.896	3.667
	Savannakhet	2.61	0.15	0.00	2.327	2.925
	Sekong	4.01	0.27	0.00	3.516	4.569
	Vientiane Capital	0.61	0.04	0.00	0.535	0.697
	Vientiane province	0.89	0.06	0.08	0.781	1.013
	Xayabouly	0.92	0.06	0.25	0.805	1.057
	Xiengkhouang	4.57	0.27	0.00	4.069	5.136
	^a^Phongsaly	1.00				

^a^Reference group

The incidence rate of death/injury was found to be significantly different between provinces. The significantly higher incidence rate was observed in the majority of the provinces such as Attapeu (IRR = 5.94, 95% CI: 5.251–6.709, *p* < 0.01), Xieng Khouang (IRR = 4.57, 95% CI: 44.069–5.136, p < 0.01), Sekong (IRR = 4.01, 95% CI: 3.516–4.569, *p* < 0.01), Oudomxay (IRR = 3.27, 95% CI: 2.898–3.681, *p* < 0.01), Saravan (IRR = 3.26, 95% CI: 2.896–3.667, *p* < 0.01), Savannakhet (IRR = 2.61, 95% CI: 2.327–2.925, *p* < 0.01), Bokeo (IRR = 3.3, 95% CI: 2.873–3.796, *p* < 0.01), Huaphan (IRR = 2.52, 95% CI: 2.2.237–2.835, *p* < 0.01), Luang Namtha (IRR = 2.27, 95% CI: 1.987–2.594, *p* < 0.01), Khammuane (IRR = 1.94, 95% CI: 1.718–2.187, *p* < 0.01), and Luang Prabang (IRR = 1.69, 95% CI: 1.1.504–1.908, *p* < 0.01) while the incidence rate was lower in Champasack and Vientiane Capital (Table 3).

As show in Table 4, scrap collection (OR = 2.30, 95% CI: 1.073–4.943, *p* = 0.03) was associated with higher odds of death compared to being a bystander at the time of the incident, while animal disturbance (OR = 0.65, 95% CI: 0.566–0.757, *p* < 0.01), making a fire (OR = 0.46, 95% CI: 0.403–0.533, p < 0.01), tampering with a device (OR = 0.51, 95% CI: 0.438–0.594, *p* < 0.01), travelling (OR = 0.80, 95% CI: 0.728–0.881, *p* < 0.01), warfare (OR = 0.66, 95% CI: 0.604–0.735, *p* < 0.01), and farming and gardening (OR = 0.82, 95% CI: 0.735–0.920, *p* < 0.01) were associated with lower odds of death during 1965–1979. The case fatality was significantly associated with device type. Big bomb (OR = 1.38, 95% CI: 1.243–1.522, *p* < 0.01) was 1.38 times more likely than landmines to result in death while bombie, grenade, mortar, fuse and white phosphorus were less likely to result in death. The case fatality was significantly different between provinces. During this period, a significantly higher odds of death was observed in Attapeu (OR = 2.04, 95% CI: 1.579–2.630, *p* < 0.01), Saravan (OR = 1.54, 95% CI: 1.205–1.961, *p* < 0.01), Savannakhet (OR = 1.79, 95% CI:, 1.419–2.276, *p* < 0.01), and Sekong (OR = 1.66, 95% CI: 1.258–2.177, *p* < 0.01).

**Table 4:** Logistic regression analysis of case fatality during the conflict period (1965–1979)

		OR	*P.* value	95% CI	
Gender	Male	0.97	0.41	.903	1.043
	^a^Female	1.00			
Cause of Accident	Bombed by plane	1.02	0.63	0.919	1.147
	Collecting Food Water or Wood	0.91	0.17	0.804	1.039
	Disturbed by animals	0.65	0.00	0.566	0.757
	Gun Violence	1.12	0.05	1.002	1.264
	Making Fire	0.46	0.00	0.403	0.533
	Playing	0.84	0.10	0.683	1.038
	Scrap Collection	2.30	0.03	1.073	4.943
	Tampering	0.51	0.00	0.438	0.594
	Travelling	0.80	0.00	0.728	0.881
	Warfare	0.66	0.00	0.604	0.735
	Farming or Gardening	0.82	0.00	0.735	0.920
	Suicide	0.54	0.66	0.033	8.719
	^a^Doing nothing/ Bystander	1.00			
Uxo type	Bombie	0.84	0.00	0.769	0.918
	Artillery shell	0.89	0.02	0.817	0.980
	Big Bomb	1.38	0.00	1.243	1.522
	Small Bomb	1.07	0.37	0.918	1.252
	Grenade	0.72	0.00	0.652	0.793
	Mortar	0.75	0.00	0.667	0.839
	Rocket	0.96	0.55	0.849	1.092
	Fuse	0.41	0.00	0.282	0.594
	White Phosphor Bomb	0.67	0.02	0.471	0.948
	Other	0.97	0.57	0.895	1.062
	Unknown	1.69	0.00	1.487	1.939
	^a^Landmine	1.00			
Provinces	Attapeu	2.04	0.00	1.579	2.630
	Bokeo	1.07	0.62	0.807	1.427
	Bolikhamxay	0.83	0.17	0.629	1.085
	Champasack	1.11	0.44	0.854	1.436
	Huaphan	1.27	0.05	0.995	1.617
	Khammuane	1.21	0.14	0.940	1.549
	Luang Namtha	1.11	0.44	0.845	1.462
	Luang Prabang	0.95	0.67	0.744	1.209
	Oudomxay	1.25	0.08	0.973	1.605
	Saravan	1.54	0.00	1.205	1.961
	Savannakhet	1.79	0.00	1.419	2.276
	Sekong	1.66	0.00	1.258	2.177
	Vientiane Capital	0.33	0.00	0.253	0.443
	Vientiane province	0.96	0.78	0.738	1.256
	Xayabouly	0.76	0.05	0.572	0.998
	Xiengkhouang	0.98	0.89	0.774	1.249
	^a^Phongsaly	1.00			

^a^Reference group

### Strata 2: Post-conflict (1980–2008)

The Poisson regression model indicated that compared to females, males had an incidence rate 4.6 times greater (IRR = 4.64, 95% CI: 4.354–4.942, p < 0.01). Gun violence (IRR = 2.39, 95% CI: 2.205–2.583, *p* < 0.01), warfare (IRR = 2.45, 95% CI: 2.277–2.646, *p* < 0.01), travelling (IRR = 1.59, 95% CI: 1.470–1.716, *p* < 0.01), and farming/gardening/digging (IRR = 1.02, 95% CI: 0.954–1.097, *p* < 0.01) were physical activities at the time of incident significantly associated with casualty rate. Scrap collection and suicide had an incidence rate of 0.3 times and 0.27 times lower than being a bystander at the time of the incident, respectively. Bombies, compared to landmines had an incidence rate 1.07 times greater for the incident (death/injury) while the other recorded explosive devices were associated with a lower incidence rate (refer Table 5).

**Table 5:** Poisson regression analysis of case fatality during the post-conflict period (1980–2008)

		IRR	Std. Err.	p-value	95% CI	
Gender	Male	4.64	0.15	0.00	4.354	4.942
	^a^Female	1.00				
Cause of Accident	Bombed by plane	0.90	0.10	0.36	0.719	1.126
	Collecting Food Water or Wood	0.97	0.04	0.40	0.893	1.046
	Disturbed by animals	0.71	0.04	0.00	0.643	0.784
	Gun Violence	2.39	0.10	0.00	2.205	2.583
	Making Fire	0.90	0.04	0.01	0.833	0.979
	Playing	0.78	0.04	0.00	0.712	0.852
	Scrap Collection	0.30	0.03	0.00	0.247	0.358
	Tampering	0.70	0.04	0.00	0.627	0.785
	Travelling	1.59	0.06	0.00	1.470	1.716
	Warfare	2.45	0.09	0.00	2.277	2.646
	Farming or Gardening/digging	1.02	0.04	0.53	0.954	1.097
	Suicide	0.27	0.09	0.00	0.144	0.500
	^a^Bystander/Doing nothing					
Uxo type	Bombie	1.07	0.03	0.03	1.006	1.143
	Artillery shell	0.64	0.02	0.00	0.593	0.687
	Big Bomb	0.24	0.02	0.00	0.203	0.295
	Small Bomb	0.33	0.03	0.00	0.282	0.396
	Grenade	0.40	0.02	0.00	0.372	0.436
	Mortar	0.43	0.02	0.00	0.393	0.468
	Rocket	0.56	0.03	0.00	0.503	0.615
	Fuse	0.36	0.04	0.00	0.298	0.446
	White Phosphor Bomb	0.15	0.03	0.00	0.110	0.210
	Other	0.78	0.03	0.00	0.731	0.831
	Unknown	0.68	0.03	0.00	0.626	0.742
	^a^Landmine	1.00				
Provinces	Attapeu	3.52	0.37	0.00	2.857	4.332
	Bokeo	1.54	0.18	0.00	1.219	1.950
	Bolikhamxay	1.13	0.12	0.25	0.917	1.396
	Champasack	0.47	0.05	0.00	0.378	0.579
	Huaphan	1.83	0.19	0.00	1.504	2.239
	Khammuane	1.72	0.18	0.00	1.408	2.099
	Luang Namtha	1.20	0.14	0.12	0.954	1.513
	Luang Prabang	1.66	0.17	0.00	1.350	2.035
	Oudomxay	1.93	0.21	0.00	1.563	2.381
	Saravan	1.13	0.12	0.25	0.918	1.386
	Savannakhet	1.51	0.15	0.00	1.251	1.832
	Sekong	1.72	0.20	0.00	1.375	2.146
	Vientiane Capital	0.47	0.06	0.00	0.372	0.604
	Vientiane province	0.63	0.07	0.00	0.508	0.793
	Xayabouly	0.97	0.10	0.78	0.786	1.199
	Xiengkhouang	2.56	.25	0.00	2.12	3.112
	^a^Phongsaly	1.00				

^a^Reference group

The incident (death/injury) significantly differed between provinces. The highest incidence rate was observed in Attapeu (IRR = 3.52, 95% CI: 2847–4.332, *p* < 0.01), Xieng Khouang (IRR = 2.56, 95% CI: 2.12–3.112, *p* < 0.01), Oudomxay (IRR = 1.93, 95% CI: 1.563–2.381, *p* < 0.01), Sekong (IRR = 1.72, 95% CI: 1.375–2.146, *p* < 0.01), Huaphan (IRR = 1.83, 95% CI: 1.504–2.239, *p* < 0.01), Khammuane (IRR = 1.72, 95% CI: 1.408–2.099, *p* < 0.01), Bokeo (IRR = 1.54, 95% CI: 1.219–1.950, p < 0.01) while the incidence rate was lower in Champasack, Vientiane Capital, and Vientiane province (Table 5).

The logistic regression analysis indicated a statistical significant difference in the odds of deaths between male and female. The odds of death were 1.14 times higher in males than in females (Table 4). Gun violence (OR = 1.35, 95% CI: 1.134–1.602, *p* < 0.01) and suicides (OR = 11.56, 95% CI: 1.493–89.631, *p* = 0.01) were associated with higher odds of death compared to being a bystander at the time of the incident, while collecting food water or wood (OR = 0.59, 95% CI: 0.499–0.703, *p* < 0.01), making a fire (OR = 0.31, 95% CI: 0.260–0.367, *p* < 0.01) and playing (OR = 0.63, 95% CI: 0.523–0.0.770, *p* < 0.01) were associated with lower odds of death post-conflict. Unlike the war period, the case fatality during this period was not significantly associated with device type. The case fatality was significantly different between provinces. The odds of death were higher in all provinces except Hauphan, Luang Prabang, Oudomxay, Vientiane Capital, Xayabouly, and Xieng Khouang (Table 6).

**Table 6:** Logistic regression analysis of case fatality during the post-conflict period (1980–2008)

Outcome		OR	*P.* value	95% CI	
Gender	Male	1.14	0.039	1.006	1.303
	^a^Female	1.00			
Cause of Accident	Bombed by plane	1.04	0.85	0.660	1.651
	Collecting Food Water or Wood	0.59	0.00	0.499	0.703
	Disturbed by animals	0.67	0.00	0.550	0.833
	Gun Violence	1.35	0.00	1.134	1.602
	Making Fire	0.31	0.00	0.260	0.367
	Playing	0.63	0.00	0.523	0.770
	Scrap Collection	1.02	0.90	0.689	1.526
	Tampering	0.71	0.00	0.569	0.885
	Travelling	0.98	0.81	0.834	1.152
	Warfare	0.81	0.01	0.687	0.956
	Farming or Gardening	0.74	0.00	0.635	0.856
	Suicide	11.56	0.01	1.493	89.631
	^a^Doing nothing/ Bystander				
UXO type	Bombie	0.93	0.32	0.811	1.071
	Artillery shell	0.76	0.00	0.648	0.892
	Big Bomb	1.09	0.62	0.774	1.541
	Small Bomb	0.92	0.64	0.647	1.311
	Grenade	0.77	0.00	0.651	0.909
	Mortar	0.67	0.00	0.552	0.811
	Rocket	0.65	0.00	0.515	0.813
	Fuse	0.20	0.00	0.121	0.341
	White Phosphor Bomb	0.33	0.00	0.148	0.751
	Other	0.62	0.00	0.540	0.713
	Unknown	0.96	0.70	0.780	1.178
	^a^Landmine	1.00			
Provinces	Attapeu	2.43	0.00	1.537	3.855
	Bokeo	1.89	0.01	1.132	3.163
	Bolikhamxay	1.62	0.04	1.032	2.570
	Champasack	1.87	0.00	1.184	2.947
	Huaphan	1.41	0.12	0.910	2.175
	Khammuane	2.04	0.00	1.313	3.183
	Luang Namtha	1.91	0.01	1.167	3.136
	Luang Prabang	0.97	0.90	0.616	1.529
	Oudomxay	0.94	0.81	0.589	1.516
	Saravan	2.00	0.00	1.286	3.124
	Savannakhet	2.53	0.00	1.662	3.866
	Sekong	1.86	0.01	1.157	3.007
	Vientiane Capital	0.33	0.00	0.181	0.604
	Vientiane province	1.780	0.01	1.119	2.832
	Xayabouly	0.86	0.53	0.547	1.368
	Xiengkhouang	1.41	0.11	0.921	2.152
	^a^Phongsaly	1.00			

^a^Reference group

## Discussion

The analysis reveals that most landmine and ERW injuries occurred during the war, and injuries remained high in the immediate post-conflict period as people returned to work on their land. This study aligns with a previously published analysis of this dataset which revealed that most casualties were male soldiers (45%) and farmers (35%) [9]. Fatal ERW injury decreased over time, and is likely to be due to improved access to health care in the post-conflict period.

The highest odds of death during the conflict period were associated with big bombs, which likely relates to the explosive force of these weapons. Landmine injuries were more common during the conflict phase (1964–1979), and it is likely that due to the severity of the injuries and limited access to appropriate care, most landmine fatalities were due to blood loss requiring immediate medical attention. Previously conducted research [9] highlights a number of other factors that may explain the high number of landmine casualties during this period: firstly, this device type may have included aerial dropped gravel mines which would be located in a similar, random spread to cluster munitions (UXO), secondly, landmines were used during ground battles and thirdly, it is likely an unknown number of respondents may have automatically stated the incident involved a landmine regardless of device type [9].

Post-conflict, gunshot wounds and suicide were associated with the highest odds of death, and may relate to the more deliberate actions of this form of explosive violence in comparison to activities such as collecting food or water, or playing that result in unintentional deaths and/or injuries. The higher incidence of warfare as a cause of death or injury post-conflict suggests some issues with the data, and it is unclear if the inclusion of warfare during this period is due to misclassification, ongoing low-level conflict, criminal activities, or reflects deaths due to historical injuries arising from the earlier conflict period. Furthermore, warfare was associated with a lower odds of death during the active conflict phase. It is unclear if this was due to an underreporting of these deaths, or that warfare-related injuries sustained during this period received some form of medical intervention that improved survivability, at least in the interim. Post conflict, bombies had the highest incidence rate for death or injury, which is unsurprising given the levels of cluster munitions contamination that has been established within Lao PDR [26].

This study shows variance between the provinces of Lao PDR with, for example, lower incidence rates recorded in Vientiane capital city and province. This is likely related to the geographical distance of Vientiane from areas heavily targeted during the war (e.g., Ho Chi Minh Trail), and the better accessibility of healthcare facilities in Vientiane compared to most of the provinces.

For the whole period included in the study, males had a higher incidence rate than females. This pattern has been documented in other contexts and relates to the gendered roles of males and females [1, 7, 27, 28]. Males typically play more active roles during conflicts and are more likely to remove UXO from areas where community members and womenfolk interact. The incidence of males experiencing death or injury during the conflict phase was 4.34 times greater than females in this study. Post conflict, males are more likely to deliberately move or try to defuse UXO, often in order to sell the metal casing for the scrap metal or fashion the metal into useful household implements, but also to make the environment safer for women and children [29, 30]. Despite often being seen as a common cause of post-conflict UXO injuries, when looked at in the post conflict period, collecting war scrap metal had the second lowest incidence rate [30]. This is probably because unlike subsistence farming activities, the collection of war scrap metal is largely seasonal and demand driven, and over time, scrap metal dealers may have learnt from experience strategies to mitigate risk when collecting remnants [29, 30].

The increase observed in non-fatal health outcomes has implications for medical and vocational rehabilitation needs and further research is warranted to better understand disabling conditions associated with ERW injuries. Further, while pressure activated landmines have been shown in adult populations to result in lower limb amputations, much less is known about on-going impairments and rehabilitation needs of UXO injuries [16] including other potential disabilities such as vision or hearing impairments [9].

No information was available related to length of stay in hospital which would provide an indication of health service costs and out-of-pocket health expenditure and lost production costs experienced by individuals and families. Both fatal and non-fatal injuries in the Lao PDR, however, can translate into significant livelihood vulnerabilities due to limited access to formal social insurance mechanisms. This is particularly pertinent in rural areas where most farmers maintain a subsistence or semi-subsistence lifestyle [31].

As with all studies, this study has some limitations. Firstly, the data we used were based on a retrospective population survey and we cannot discount the possibility that the survey failed to capture all casualties. Recall bias is of concern given in some cases the large time-lag between when the incident occurred and when the data was collected, and because in some cases the information on the incident was not collected from the casualty. Partly, because of this, the dataset also contained missing data that limited interpretation of the epidemiological patterns and risk factors for injury. In addition, we identified travel time to definitive medical care, and the provision of pre-hospital care, as likely to be important variables in determining injury outcome but were unable to include this in the regression models as these data were not collected in the survey. These limitations associated with the quality of the data are commonly found within low-resource environments, and were further compounded by issues including a lack of standardized spelling and translation, together with limited active injury surveillance meaning injury data is not prospectively captured. This highlights a need for injury surveillance systems to be implemented when mine action services are first initiated within a post-conflict context.

Nevertheless, the study had large coverage (95% of all villages in the country) and population-based studies provide more reliable information than health facility based studies because these represent the whole population rather than only those people who have sought health care. This is particularly important in resource-constrained environments such as the Lao PDR, where access to care in the case of traumatic injury is limited. Another limitation is that population figures were from 1985 and census data from 1995 meaning that the population denominators that was used in calculating rates were not necessarily accurate but was the earliest data available. Finally, the quality of the data did not allow for in-depth examination of the health consequences of ERW.

Cluster munitions continue to be used including in Iraq, Yemen and Syria and there is an urgent need for more research of the health impacts of these weapons including epidemiologic population-based surveys in affected areas [16]. There is also a need for enhanced reporting systems that coordinate with national health systems and integrate UXO injuries with broader efforts to improve injury surveillance systems [6]. In addition, health professionals and others should continue to advocate for full implementation of the Mine Ban Treaty and the Convention on Cluster Munitions.

## Conclusion

To conclude, landmine and ERW injuries have serious social and economic impact, and typically affect those who are already disadvantaged [7, 16, 17, 19, 32]. In Lao PDR, ERW, particularly cluster munitions, continue to pose a threat to human well-being and development long after the cessation of the conflict. Whilst further research is required to better document and understand the health and socio-economic consequences of landmine and ERW injuries, this study provides insight into the geographical regions and population subgroups experiencing elevated casualty numbers and odds of death. This information is vital for efficient and effective planning services that target those most vulnerable within Lao PDR. Furthermore, while injuries are decreasing, there is also a need for continued education, especially for civilians, on the dangers of landmines and ERW to help mitigate risk in affected communities.

## Abbreviations

ERW: Explosive remnants of war
Lao PDR: Lao People’s Democratic Republic
NRA: National Regulatory Authority
UXO: Unexploded ordnance

## Footnotes

1 Explosive remnants of war (ERW) refers to the explosive remnants which remain in the environment following the armed conflict and include, for example, cluster munitions, grenades, and other unexploded ordnances.
